# The Acute Flaccid Paralysis (AFP) Surveillance System in Yemen, 2010-2015: Descriptive Study Based on Secondary Data Analysis

**DOI:** 10.2196/14413

**Published:** 2019-12-06

**Authors:** Khaled Abdullah Almoayed, Ali Bin Break, Mutahar Al-Qassimi, Ali Assabri, Yousef Khader

**Affiliations:** 1 Yemen Field Epidemiology Training Program Ministry of Public Health and Population Sana'a Yemen; 2 Acute Flaccid Paralysis Surveillance Program Ministry of Public Health and Population Sana'a Yemen; 3 Yemen Field Epidemiology Training Program Community Medicine & Public Health Sana’a University Sana’a Yemen; 4 Faculty of Medicine Jordan University of Science & Technology Amman Jordan

**Keywords:** evaluation, acute, flaccid, paralysis, surveillance, Yemen

## Abstract

**Background:**

Acute flaccid paralysis (AFP) surveillance is an essential strategy for poliovirus eradication.

**Objective:**

This study aimed to evaluate the performance of the AFP surveillance system in Yemen from 2010 to 2015, identify components that require strengthening, and compare the indicators by year and governorates.

**Methods:**

This descriptive study was based on secondary analysis of AFP surveillance data reported during 2010-2015 from all Yemeni governorates. The World Health Organization (WHO) minimum performance standards were used to evaluate the performance of the AFP surveillance system.

**Results:**

A total of 3019 AFP cases were reported between January 2010 and December 2015. At the national level, AFP surveillance achieved WHO targets throughout the evaluating period for the nonpolio AFP rate of cases per 100,000 members of the population younger than 15 years of age, proportion of AFP cases reported within 7 days, proportion of AFP cases investigated within 48 hours of notification, proportion of AFP cases with two adequate stool specimens, and proportion of stool specimens from which nonpolio enterovirus was isolated. However, the proportion of specimens that arrived at the central level within 3 days of the first sample collection and the proportion of stool specimens with results sent from the reference laboratory within 28 days of receipt did not reach targets in 2011 and 2015, respectively.

**Conclusions:**

The AFP surveillance system in Yemen has met most of the WHO indicator levels. Nevertheless, the evaluation showed areas of weakness regarding the arrival of specimens at the central level within 3 days of the first sample collection and delays in processing of the results and submitting feedback by the laboratory. Therefore, there is a need to strengthen the follow-up of specimens submitted to the laboratory.

## Introduction

### Background

Poliomyelitis is a highly infectious disease caused by an enterovirus that is transmitted through the fecal-oral route or, less frequently, by a common vehicle. It affects the nervous system and can cause total paralysis in a matter of hours [1]. Paralytic polio occurs in less than 1% of children infected with poliovirus. The paralysis can cause weakness of the limbs, usually in the legs, and while some patients subsequently recover, around two-thirds suffer permanent paralysis [2]. Among those paralyzed, 5%-10% die when their breathing muscles become immobilized [1]. Polio mainly affects children under 5 years of age. Polio cases have decreased by over 99% since 1988, from an estimated 350,000 cases in more than 125 endemic countries to 74 reported cases in 2015. Recently, polio has become endemic in three countries: Afghanistan, Pakistan, and Nigeria [1,3]. There are three types of polio virus: type 1, type 2, and type 3. Cases of type 3 dropped to the lowest level, where the last case was reported in November 2012 in Nigeria [1].

Acute flaccid paralysis (AFP) is a clinical syndrome characterized by rapid onset of weakness, including weakness of respiratory function and swallowing, progressing to maximum severity within several days to weeks. This weakness is considered flaccid (ie, no spasticity) because the lesion affects the lower motor neuron, decreasing muscle tone and diminishing tendon reflex [4,5]. Because poliomyelitis causes AFP, highly sensitive surveillance for AFP with immediate case investigation and specimen collection are critical for the detection of wild poliovirus circulation, with the ultimate objective of polio eradication. AFP surveillance is also critical for polio-free certification [4,6,7].

### Acute Flaccid Paralysis Surveillance in Yemen

Yemen is located in the Middle East on the southern tip of the Arabian Peninsula, bordered on the west side by the Red Sea, on the north side by Saudi Arabia, on the south side by the Arabian Sea, and on the northeast side by Oman. The area is 527,970 km^2^ in size. In 2016, the total population of Yemen was about 26 million and the population of children less than 15 years of age was estimated to be 13.3 million. Yemen is divided into 22 governorates and 333 districts. The AFP surveillance system covers all 22 governorates, 333 districts, and about 1857 reporting sites, including the main hospitals and health centers in the country.

The AFP surveillance system was launched in Yemen in 1998 as part of the polio eradication initiative announced by the World Health Organization (WHO) in 1988. The last poliovirus outbreak in Yemen was in February 2005, when 479 cases of wild polio were reported during that outbreak; the last case reported was in February 2006. Yemen had three different outbreaks of circulating vaccine-derived polio viruses (VDPV) since April 2011: one type 2 VDPV and two separate emergencies of type 3 VDPV outbreaks [8].

### Evaluation Rationale

Regular analysis of data generated from an AFP surveillance system is important in evaluating and improving the performance of the system. This ensures optimal performance of the system and guarantees timely detection of wild poliovirus reimportation. The WHO has developed a set of performance indicators in order to ensure that AFP surveillance is adequately conducted to accurately guide the polio eradication initiative [4,6,7]. To our knowledge, the AFP surveillance system has not yet been evaluated at the central level; it was evaluated only in the Ibb and Hadhramout governorates [9,10]. This study aimed to evaluate the performance of the AFP surveillance system in Yemen from 2010 to 2015, identify components that require strengthening, and compare the indicators by year and governorates.

## Methods

This descriptive study was based on secondary data of AFP surveillance from all Yemeni districts and governorates over 6 years: 2010-2015. WHO standard indicators were used as reference to evaluate the performance of the AFP surveillance system [4].

The following seven WHO standard indicators were used:

1. Annualized nonpolio AFP rate: ≥2 AFP cases/100,000 members of the population under 15 years of age.
2. Adequacy of stool specimens: proportion (≥80%) of AFP cases with two adequate stool specimens.
3. Timeliness of case notification: proportion (≥80%) of AFP cases reported within 7 days.
4. Timeliness of case investigation: proportion (≥80%) of AFP cases investigated within 48 hours of notification.
5. Timeliness of specimen's arrival at the central level: proportion (≥80%) of specimens that arrived at the central level less than 3 days from first sample collection.
6. Nonpolio enterovirus isolation rate: proportion (≥10%) of stool specimens from which nonpolio enterovirus was isolated.
7. Timeliness of specimen processing in the laboratory: proportion (≥80%) of stool specimens with results sent from Naval Medical Research Unit Three (NAMRU-3) less than 28 days after receipt.

Data were analyzed using Microsoft Excel software. Descriptive analyses were conducted to describe the epidemiology of AFP in Yemen and to generate statistics based on the standard WHO-specified performance indicators for AFP surveillance.

## Results

### Distribution of Acute Flaccid Paralysis Cases

**Figure 1:**
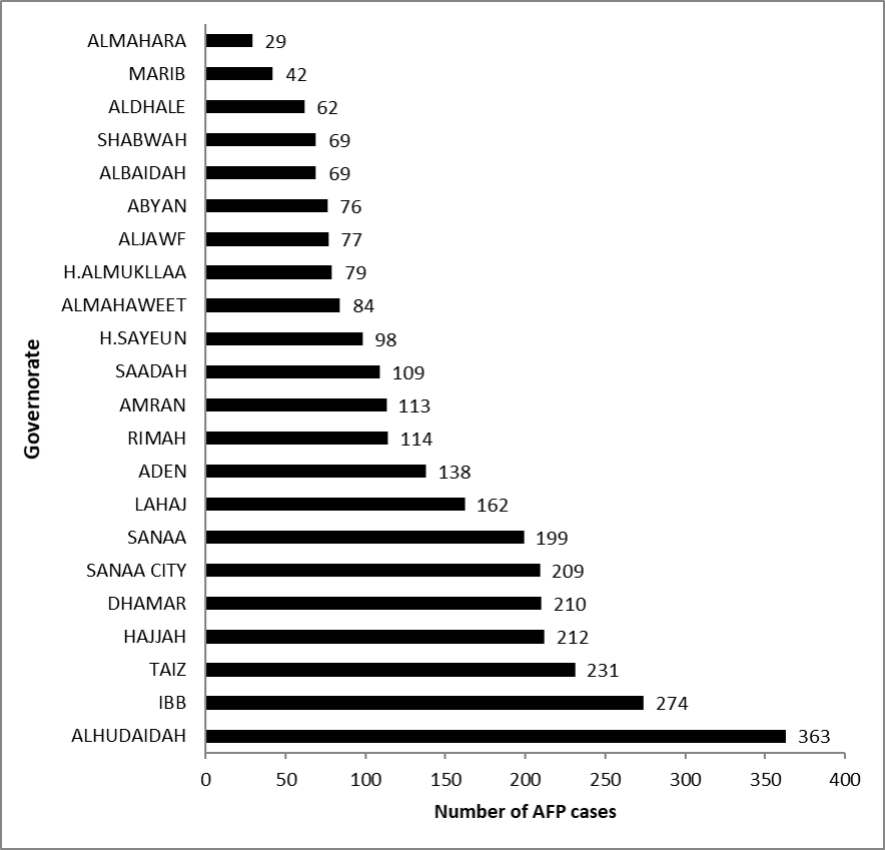
Distribution of acute flaccid paralysis (AFP) cases by governorate in Yemen from 2010 to 2015.

**Figure 2:**
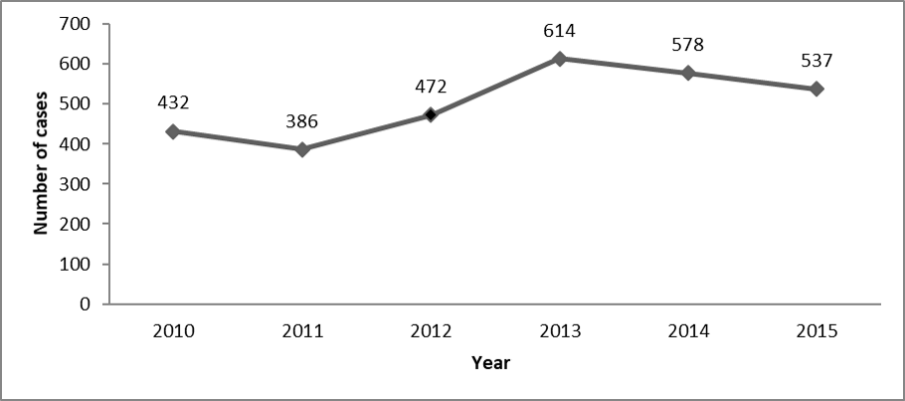
Distribution of acute flaccid paralysis (AFP) cases per year in Yemen from 2010 to 2015.

A total of 3019 AFP cases were reported between January 2010 and December 2015. Most cases were from Alhudaidah (363/3019, 12.02%) and Ibb (274/3019, 9.08%), while Almahara had the lowest number of cases (29/3019, 0.96%). Figure 1 shows the distribution of AFP cases by governorate in Yemen from 2010 to 2015. A total of 20.34% of cases (614/3019) were reported in 2013 and 12.79% (386/3019) were reported in 2011 (see Figure 2).

### Performance Indicators at the National Level

Table 1 shows the performance indicators of the AFP surveillance system in Yemen from 2010 to 2015. All performance indicators of the AFP surveillance system reached the WHO standard indicator levels in 2010, 2012, and 2014. In 2011, the proportion of specimens that arrived at the central level less than 3 days from the first sample collection was below the WHO standard indicator of 80%. During this year, this indicator level was achieved only in 75% of specimens. In addition, the proportion of stool specimens with results sent from the laboratory less than 28 days after receipt was 76% in 2013 and 48% in 2015, which are lower than the WHO standard indicator of 80%.

**Table 1:** Performance indicators of the acute flaccid paralysis (AFP) surveillance system, Yemen, 2010-2015.

Indicator	Target	Year					
		2010	2011	2012	2013	2014	2015
Nonpolio AFP detection rate/100,000 population members under 15 years of age, number of cases	≥2	3.9	3.4	4.0	5.1	4.6	4.1
Proportion of AFP cases reported within 7 days, %	≥80	89	85	84	82	86	85
Proportion of AFP cases investigated within 48 hours of notification, %	≥80	100	100	99	100	98	99
Proportion of AFP cases with two adequate stool specimens, %	≥80	97	91	93	91	95	91
Proportion of specimens that arrived at the central level within 3 days of first sample collection, %	≥80	92	75	91	87	87	85
Proportion of stool specimens from which nonpolio enterovirus was isolated, %	≥10	17	16	22	17	18	18
Proportion of stool specimens with results sent from laboratory within 28 days of receipt by laboratory, %	≥80	84	85	88	76	83	48

### Performance Indicators at the Governorate Level

#### Nonpolio Acute Flaccid Paralysis Detection Rate per 100,000 Members of the Population Under 15 Years of Age

**Figure 3:**
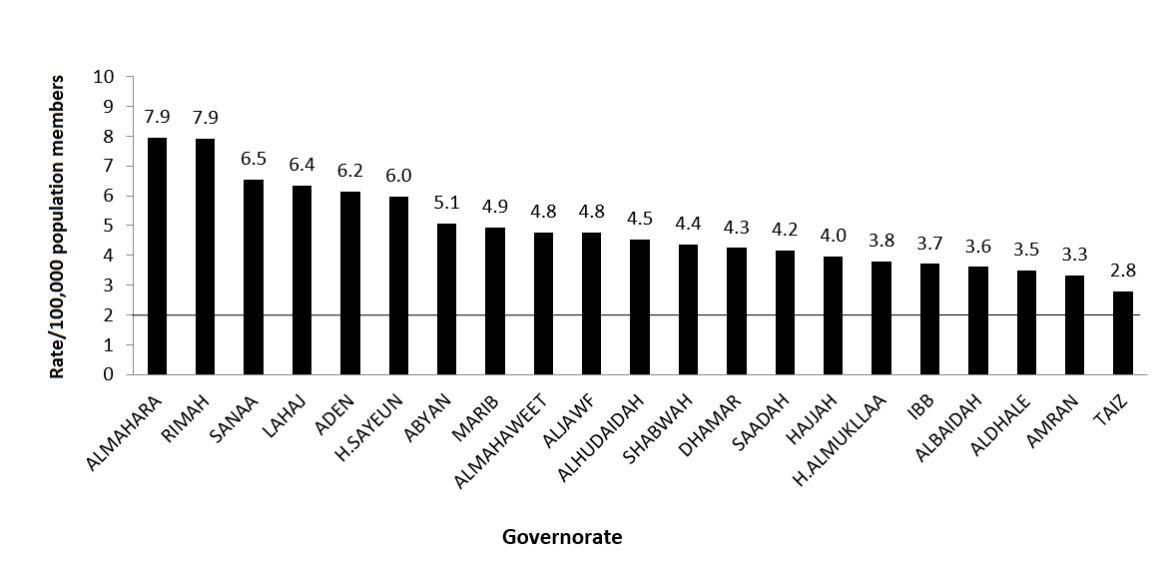
Average nonpolio acute flaccid paralysis (AFP) rate/100,000 members of the population by governorate in Yemen from 2010 to 2015.

The nonpolio AFP detection rate in all governorates met the WHO standard indicator level of ≥2 AFP cases per 100,000 members of the population under 15 years of age during the sixth year. Both Almahara and Rimah governorates had the highest nonpolio AFP rates while Taiz had the lowest rate (see Figure 3).

#### Proportion of Acute Flaccid Paralysis Cases Investigated Within 48 Hours of Notification

Similar to nonpolio AFP detection rate, the percent of AFP cases that were investigated within 48 hours of notification met the WHO standard indicator level of ≥80% in all governorates during the evaluation years.

#### Adequacy Rate of Stool Specimens

A total of 17 out of 22 governorates (77%) achieved the WHO standard indicator level (≥80%) of proportion of AFP cases with two adequate stool specimens. A total of 5 governorates (23%) failed to achieve this indicator level, namely, Aldhalae, Abyan, Alhudaidah, Amran, and Dhamar.

#### Proportion of Acute Flaccid Paralysis Cases Notified Within 7 Days

Only 9 out of 22 governorates (41%) notified officials of AFP cases within 7 days at the WHO standard indicator rate of ≥80% during the evaluation years.

#### Proportion of Stool Specimens From Which Nonpolio Enterovirus Was Isolated

Aljawf and Hajjah were the only governorates that met the WHO standard indicator level of ≥80% of stool specimens from which nonpolio enterovirus was isolated during all evaluation years. Although Alhudaidah, Lahj, and Sana'a governorates failed to reach the WHO standard indicator level in 1 year, they were considered the top 3 governorates regarding this indicator, following Aljawf and Hajjah.

#### Proportion of Specimens That Arrived at the Central Level Less Than 3 Days of the First Sample Collection

The proportion of specimens that arrived at the central level within 3 days from the first sample collection reached the WHO standard indicator level (≥80%) in 7 of the 22 governorates (32%) during all evaluation years. These governorates were Aldhalae, Hadhramout Sayeun, Lahj, Rimah, Sa'adah, Sana'a, and Taiz. The other 15 governorates failed to achieve this indicator level in some years.

#### Proportion of Stool Specimens With Results Sent From the Laboratory Less Than 28 Days After Receipt

**Figure 4:**
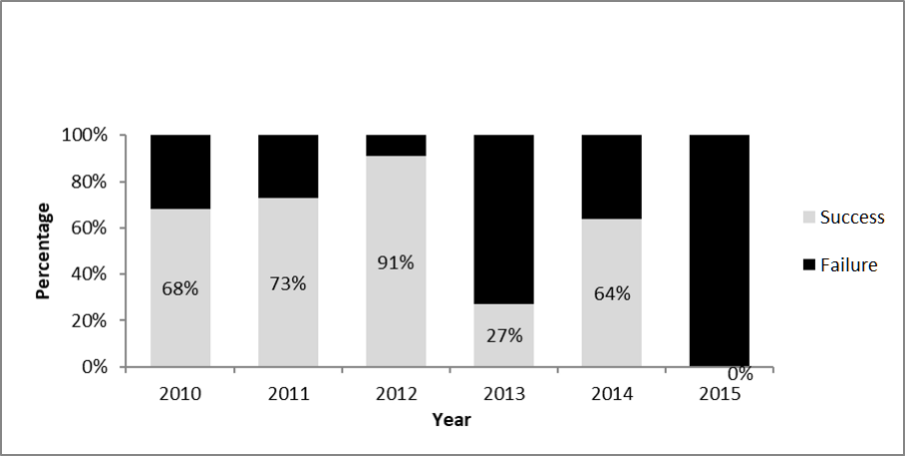
Percent of governorates that received laboratory results less than 28 days after receipt by the laboratory in Yemen from 2010 to 2015.

In 2015, this WHO standard indicator level was not achieved by any of the governorates. In 2013, this indicator level was achieved by only 5 out of 22 (23%) governorates (see Figure 4).

## Discussion

### Principal Findings

During the study period, Yemen was facing a threat of polio reintroduction from countries where the virus was still circulating [8]. A strong AFP surveillance system is critical for the early detection of wild poliovirus circulation, with the ultimate objective of polio eradication. The findings of this study showed that the nonpolio AFP detection rate reached the WHO standard indicator level (≥2 AFP cases/100,000 members of the population under 15 years of age) throughout the study period. Furthermore, on disaggregating the nonpolio AFP rates by governorates, all governorates performed well. These results indicate that the AFP surveillance system was sensitive and able to detect any polio case reimported into the country during the study period [4,7]. Compared with other studies in different countries and periods, the nonpolio AFP rate in Yemen was similar to that in Sierra Leone (2005-2012) [11] and Akwa Ibom State, Nigeria (2004-2009) [12], and higher than that in Iraq (1997-2011) [13], Hamdan, Iran (2002-2009) [14], South Africa (2005-2009) [15], Italy (1997-2007) [16], and Brazil (1990-2000) [17].

Increasing the proportion of AFP cases notified within 7 days increases the chance of timely investigation and response needed to prevent further transmission of infection. This study showed that the proportion of AFP cases notified within 7 days in Yemen was slightly higher than the WHO standard of ≥80% throughout the study period. This finding is better than that from Iraq (1997-2011) [13]. The positive performance of this indicator indicates that there would be a better chance of collecting stool specimens within 14 days.

The AFP surveillance system succeeded in investigating more than 98% of AFP cases within 48 hours of notification at the national level and more than 96% at the governorate level, exceeding the WHO standard indicator level of ≥80%. These proportions are higher than the reported proportions from South Africa and Iraq and are similar to the proportions reported in Akwa Ibom State, Nigeria [12,15]. When investigating AFP cases, many important data are collected, such as notification data, clinical case history, and vaccination status; collection of two adequate stool specimens also occurs at this time.

The proportion of AFP cases with collection of two adequate stool specimens is an important indicator for any AFP surveillance system. In this study, Yemen's AFP surveillance system exceeded this indicator level throughout the study period. The rate of adequate specimens collected in Yemen was 93% of total cases. This rate was high when compared to that in many countries around the world. However, 5 out of 22 (23%) governorates were not strong in this indicator. A total of 13 out of 22 (59%) governorates that failed to surpass the WHO standard indicator level (≥80%) of AFP cases notified within 7 days were able to reach the WHO standard indicator level (≥80%) of AFP cases with collection of two adequate stool specimens, except the Alhudaidah governorate in 2013.

At the national level, the proportion of specimens that arrived at the central level within 3 days of the first sample collection reached the WHO standard indicator level (≥80%) during all evaluation years except 2011 (75%), when a political revolution occurred. Because of that revolution, the internal situation of the country deteriorated, the main roads were closed, and fuel supply deliveries decreased and sometimes stopped. As a result, the proportion of specimens that arrived at the central level within 3 days of the first sample collection decreased dramatically from 2010 (92%) to 2015 (85%). This performance was also reflected at the governorate level, where most of the governorates failed to maintain the specimens' arrival rate above the WHO standard. Surprisingly, Sana'a City failed to achieve this indicator level for 4 years out of 6, although that was at the central level of the system.

After the arrival of stool specimens at the central level, they were sent to the NAMRU-3 laboratory in Egypt for diagnosis. Nonpolio enterovirus should be isolated from at least 10% of stool specimens received at the laboratory in order to meet WHO standards [4,7]. This indicator evaluated the quality of the reverse cold chain process and how well the laboratory was able to perform the routine isolation of enterovirus. Yemen's AFP surveillance system would have been able to achieve this indicator level throughout the study period; however, the electricity was suspended for some lengthy periods in the country, particularly in 2011. Only 2 governorates out of 22 (9%)—Aljawf and Hajjah—achieved this indicator level during the entire study period, while 20 (91%) governorates failed in some of the years: 13 governorates in 1 year, 2 governorates in 2 years, and 5 governorates in 3 years. The diagnosis of AFP cases and the feedback via results from the NAMRU-3 to Yemen's AFP surveillance system is an essential step to control and investigate polio outbreaks when they occur. The performance of the NAMRU-3 failed to fulfill the WHO standard indicator level (≥80%) regarding sending back results within 28 days of stool specimen receipt in 2013 and 2015. This performance standard indicator level was achieved less in Yemen than in Iraq, Nigeria, and South Africa [2,13,15].

### Conclusions

The AFP surveillance system in Yemen has met most of the WHO standard indicator levels. One of its weaknesses is the delay in receiving results from NAMRU-3 laboratory. This delay could lead to negative consequences regarding detection of poliovirus. Supporting the reference lab in Yemen in testing AFP specimens is strongly recommended.

## Abbreviations

AFP: acute flaccid paralysis
NAMRU-3: Naval Medical Research Unit Three
VDPV: vaccine-derived polio viruses
WHO: World Health Organization
